# Transcranial magnetic stimulation as a countermeasure for behavioral and neuropsychological risks of long-duration and deep-space missions

**DOI:** 10.1038/s41526-024-00401-8

**Published:** 2024-05-28

**Authors:** Afik Faerman, Derrick M. Buchanan, Nolan R. Williams

**Affiliations:** Department of Psychiatry and Behavioral Sciences, Stanford University School of Medicine, Stanford, CA USA

**Keywords:** Health care, Human behaviour

## Abstract

Long-duration spaceflight missions are on the rise. However, recent literature suggests that prolonged and deep-space exposure is likely to introduce increased risks for brain health and consequent detriments to performance and well-being. Given up-to-date evidence, we argue that transcranial magnetic stimulation (TMS) is a promising solution for mitigating behavioral and neurocognitive risks associated with long-duration and deep-space missions. We provide support from recent Earth-based applications of TMS and review several advantages it holds over current treatment approaches. Lastly, we highlight some of the needs in the process of applying such technology to the spaceflight environment.

## Introduction

These are exciting times for the global space industry; governmental and commercial space ventures have grown considerably over the past decade, and this growth is projected to expand further in the near future^1^. Human exploration of space, however, is facing new challenges that have yet to be solved. As planned and prospective missions extend both in duration and reach, the risks of human exposure to the spaceflight environment are a growing concern for crew health and well-being, as well as for mission success. Among the multisystemic risks of space exposure, the National Aeronautics and Space Administration (NASA)^2,3^ recognizes that spaceflight can compromise the central nervous system (CNS) and could lead to behavioral^4^ and neuropsychological^5^ impairments. Specifically, extended stay in isolated, confined, and extreme (ICE) environment, exposure to cosmic radiation, structural and functional alterations to the CNS in micro and zero gravity, and space-related disruption of sleep and circadian rhythms pose major threats to behavioral and cognitive health and performance of space crews^5,6^.

To address potential in-mission psychiatric needs, behavioral health countermeasures have been developed and deployed on board the International Space Station (ISS)^7^. For example, the med kit on the ISS includes a variety of psychiatric medications^8^, which come with several limitations (see Friedman and Bui^8^ for an in-depth review of specific medications). First, medications require substantial storage room, have relatively short shelf lives, and are a finite resource. These downsides limit their use in long-duration deep-space missions. Optimal storage of medication, including temperature control protection from radiation, may also be a challenge^8,9^. Additionally, for some of the medications in use, active pharmaceutical content (API) may degrade faster in space than on Earth^8,10^. Each of these factors plays a role in maintaining the potency and efficacy of the drug. Moreover, pharmacotherapies have systemic effects and may have a negative impact on performance^11^ and a relatively slow onset to treatment response of up to 6 weeks. Lastly, approximately 20–60% of people are resistant to typical pharmacotherapies^12^. It is, therefore, critical to prospectively equip crews with effective yet sustainable, non-finite solutions for treating psychological and cognitive symptoms if and when they arise. Here, we wish to advocate for transcranial brain stimulation (TMS) as a potential countermeasure for future spaceflight behavioral health.

## Transcranial magnetic stimulation

At present, TMS is already FDA-approved for major depression disorder, obsessive-compulsive disorder, pain management, migraines, and smoking cessation. Advancements in TMS research will likely lead to future FDA approvals. For example, there is already robust literature, including multiple randomized controlled trials and meta-analyses of the potential effectiveness and efficacy of TMS in treating symptoms of anxiety^13^, posttraumatic stress^14^, post-stroke recovery^15^, and some sleep disorders^16^. One of the advantages of TMS is that a single device has the potential to treat many conditions simply by changing the positioning of the coil and stimulation settings. As such, if a TMS machine is available for space crews, they will be able to receive software and protocol updates that could add applications to a given TMS machine and improve its effectiveness based on discoveries made on Earth during the mission.

A standard repeated TMS (rTMS) intervention (such as the FDA-approved protocol^17^) includes 30 three-minute sessions of rTMS over 30 days. However, recently FDA-cleared accelerated protocols in treatment-resistant depression, such as the Stanford Neuromodulation Therapy (SNT^18^), provide a much faster treatment response within five days, substantially faster than the average duration of pharmacological use until clinically meaningful effects appear (e.g., 1–6 weeks for SSRIs^19^) and superior to antidepressants in treatment effects^20^. Continuous antidepressant treatment is also recommended to minimize the risk of relapse after discontinuation (e.g., 10–12 months^21^), while periodic rTMS maintenance sessions allow remission for months and even years^22^. There is also evidence that relapse followed by retreatment with rTMS like SNT remains effective^23^. Furthermore, modern rTMS protocols such as SNT are designed with a Precision Medicine framework, utilizing baseline neuroimaging to tailor target localization and stimulation intensity for each individual based on their brain structure and function. It is possible that, in healthy individuals, prophylactic protocols for individuals presenting prodromally could be even briefer; however, as of yet, no studies have tested this hypothesis.

Naturally, funding for clinical research typically prioritizes testing treatments for more distressed and underserved populations, and opportunities to test TMS as an early preventative intervention is still needed. However, TMS can significantly enhance cognitive performance in healthy adults^24^, as well as older adults and those with cognitive impairments^25^. Furthermore, preliminary findings indicate the ability of TMS to facilitate neuroplasticity^26^. Such evidence is encouraging, as it suggests a proactive role of TMS and emphasizes the need for further research into cognitive enhancement in the general population, including healthy, high-performing individuals, and can be tested as a means to counter the deleterious effects of the space environment on cognition^5^. Accordingly, a TRISH-funded study is currently studying TMS for enhancement of cognitive as a direct countermeasure to cognitive decline in long-duration space missions^27^. For a detailed review of TMS and other noninvasive brain stimulation (NIBS) for space exploration, see Romanella et al.^28^.

## Discussion

TMS provides a promising countermeasure approach to mitigating behavioral and cognitive risks of spaceflight and offers several practical advantages over pharmacological approaches. First, TMS devices require only a fixed amount of space to be stored (compared to medication, which requires more storage as the duration of the mission increases) and offer theoretically non-finite uses. Second, TMS treatment protocols can be personalized for optimal effects for each individual brain and symptoms targeted. Furthermore, TMS is easy to automatize and target very specific neural circuits, and the same device could be used as a countermeasure for different symptoms (by adjusting the target and stimulation protocol). As novel protocols are designed, software updates and new operating instructions can be easily transmitted to space crews. Overall, TMS offers a sustainable and effective countermeasure, even in pharmacotherapy-resistant cases.

**Fig. 1:**
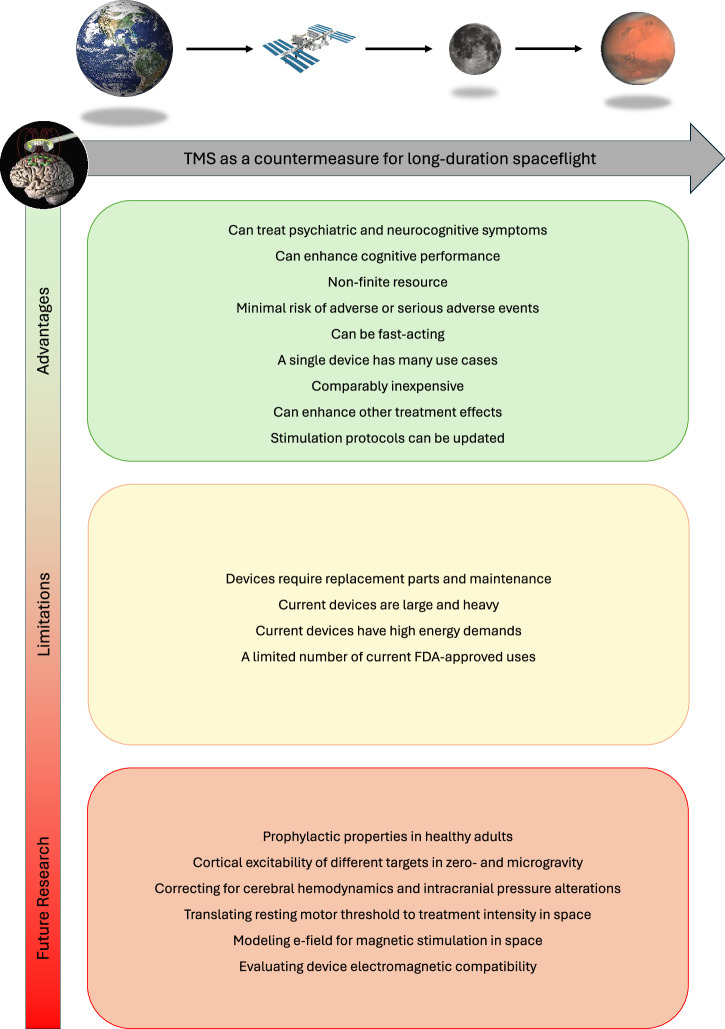
Visual summary. Advantages, limitations, and need for further research into using transcranial magnetic stimulation (TMS) in spaceflight.

Despite these strengths, further research is needed to accurately and effectively implement TMS or other NIBS for space crews (see Fig. 1 for a summary). First, data on the neuropsychiatric benefits of TMS is almost exclusively collected in clinical populations. Future studies require testing these findings in individuals with subclinical presentations; although the mechanism of action should apply in healthy individuals, supporting data is needed to substantiate these claims. Second, different space environments may cause the TMS stimulation to interact differently with the brain than it does on Earth. For example, a recent study, funded by the Translational Research Institute for Space Health (TRISH), found that due to a shift in brain positioning in the skull in microgravity, less energy is needed (12% reduction) to achieve behaviorally observable motor cortex excitation via TMS (i.e., the motor threshold for establishing TMS dosage^29^). This finding also highlights potential factors that may reduce the effectiveness of TMS in space applications and challenges to overcome^28,30^. Specifically, prolonged physical movement of the brain in the skull, changes to cerebral hemodynamics, and alterations to intracranial pressure may impact cortical excitability^29^. It is also possible that the cortical excitability of other neuronal networks changes in a different manner than the motor cortex^29,30^. For example, a previous electroencephalogram study suggested the frontal network, often targeted by clinical TMS applications, may decrease during parabolic flight^31^. As such, a more detailed delineation of excitability changes across different stimulation targets in microgravity is warranted. Additionally, further research is needed to determine the amount of variance between individuals and account for individual morphological and positional shifts of the brain in zero gravity. This is a crucial step because establishing an accurate motor threshold (i.e., stimulation dose) of TMS is critical not only for treatment efficacy but also to ensure safety. A possible solution that has been demonstrated on Earth but not yet in space or space analogs is formulating dedicated depth correction equations^32,33^. On Earth, depth correction equations can be applied to neuroimaging targets to account for individual factors such as skull thickness and fatty tissue between the scalp and the brain (specifically, the cortical TMS treatment target). It is likely that such a solution could be applied to correct shifts of the brain while under conditions of microgravity. It is also possible that varying electromagnetic field exposure in the spaceflight environment could interact with the efficacy of the magnetic stimulation. Future research may be able to simulate such factors in an e-field model leading to potential correction models (see Romanella et al.^30^ for proposed pathways to optimizing TMS use in space).

TMS is one of the most studied and clinically applied approaches of NIBS, with notable effectiveness and good safety profiles. However, other NIBS approaches may prove useful countermeasures for long-duration deep-space missions by enhancing cognitive performance during preparatory training and in-flight, improving behavioral health symptoms, and studying biomarkers of astronaut performance and recovery^28^. For example, transcranial electrical stimulation (TES), such as transcranial direct current stimulation (tDCS), has many potential similar use cases as TMS. A recent secondary meta-analysis concluded that tDCS is “definitely effective (Level A), for depression, and probably effective (Level B), for neuropathic pain, fibromyalgia, migraine, post-operative patient-controlled analgesia and pain, Parkinson’s disease (motor and cognition), stroke (motor), epilepsy, schizophrenia, and alcohol addiction”^34^ (p. 257). tDCS also has the distinct advantage of being much more portable and requires less energy than current TMS devices^28^. However, tDCS is substantially less specific than TMS and may impact a variety of collateral networks beyond the targeted one^34^. Additionally, no TES is presently approved by the FDA for any health condition.

With regard to device compatibility, two issues require further investigation. First, TMS devices will require evaluation of electromagnetic compatibility (e.g., as done in the ISS^35^). Second, TMS devices will require the development of more compact and portable modules. A proof of concept for a portable TMS has been done previously^36^, but adaptation to modern devices is warranted. Careful consideration would also need to be given to the manufacturing of highly durable devices as repairs would be difficult in space without also carrying the proper replacement parts. Such development would prove beneficial for other Earth-based settings. For example, compact devices would make NIBS a more viable option for military deployment, naval vessels, and remote, underserved communities with no access to a TMS clinic. As such, the potential market for space-adapted TMS devices will likely prove much broader than serving space crews alone (Fig. 1).

## Conclusion

Here, we argue that TMS is a logical and promising alternative or adjunct solution for mitigating behavioral and neurocognitive risks associated with long-duration and deep-space missions. To that end, further funding will be imperative for testing the effects of TMS in non-clinical populations, establishing preventative uses, and adapting TMS to the spaceflight environment. As human space exploration continues to advance into long-duration and deep-space missions, such as the Artemis and Mars missions, it is ever important to consider sustainable countermeasures that promote the performance and well-being of astronauts and overall mission success.

## Data Availability

Data sharing is not applicable to this article as no datasets were generated or analyzed.
